# Isolation and Analysis of Plasma-Derived Exosomes in Patients With Glioma

**DOI:** 10.3389/fonc.2019.00651

**Published:** 2019-07-16

**Authors:** Luz M. Cumba Garcia, Timothy E. Peterson, Mario A. Cepeda, Aaron J. Johnson, Ian F. Parney

**Affiliations:** ^1^Mayo Clinic Graduate School of Biomedical Sciences, Mayo Clinic, Rochester, MN, United States; ^2^Department of Immunology, Mayo Clinic, Rochester, MN, United States; ^3^Department of Neurological Surgery, Mayo Clinic, Rochester, MN, United States; ^4^Department of Urology, Mayo Clinic, Rochester, MN, United States; ^5^Department of Neurology, Mayo Clinic, Rochester, MN, United States

**Keywords:** glioblastoma, plasma-derived exosomes, isolation, immunosuppression, biomarkers

## Abstract

Gliomas including glioblastoma (GBM) are the most common primary malignant brain tumors. Glioma extracellular vesicles (EVs) including exosomes have biological effects (e.g., immunosuppression) and contain tumor-specific cargo that could facilitate liquid biopsies. We aimed to develop a simple, reproducible technique to isolate plasma exosomes in glioma patients. Glioma patients' and normal donors' plasma exosomes underwent brief centrifugation to remove cells/debris followed by serial density gradient ultracentrifugation (DGU). EV size/concentration was determined by nanoparticle tracking. Protein cargo was screened by array, western blot, and ELISA. Nanoscale flow cytometry analysis quantified exosome and microvesicle populations pre- and post-DGU. One-step DGU efficiently isolates exosomes for nanoparticle tracking. Wild type isocitrate dehydrogenase glioma patients' (i.e., more aggressive tumors) plasma exosomes are smaller but higher concentration than normal donors. A second DGU efficiently concentrates exosomes for subsequent cargo analysis but results in vesicle aggregation that skews nanoparticle tracking. Cytokines and co-stimulatory molecules are readily detected but appeared globally reduced in GBM patients' exosomes. Surprisingly, immunosuppressive programmed death-ligand 1 (PD-L1) is present in both patients' and normal donors' exosomes. Nanoscale flow cytometry confirms efficient exosome (<100 nm) isolation post-DGU but also demonstrates increase in microvesicles (>100 nm) in GBM patients' plasma pre-DGU. Serial DGU efficiently isolates plasma exosomes with distinct differences between GBM patients and normal donors, suggesting utility for non-invasive biomarker assessment. Initial results suggest global immunosuppression rather than increased circulating tumor-derived immunosuppressive exosomes, though further assessment is needed. Increased glioma patients' plasma microvesicles suggest these may also be a key source for biomarkers.

## Introduction

Gliomas, including glioblastoma (GBM), are the most common malignant brain tumors and are highly lethal (1). Despite aggressive treatment with surgery, radiation, and chemotherapy, GBM is nearly universally fatal within 5 years. Initial evaluation relies on magnetic resonance imaging (MRI), but tissue histopathology, immunohistochemistry, and molecular analysis (2, 3) are required for definitive diagnosis. Post-operative imaging is performed to evaluate the extent of surgical resection and tumor progression. However, MRI inadequately correlates with actual neoplastic disease burden, failing to address the micro-infiltrative disease beyond the borders of radiological depiction (4–6). Furthermore, MRI can be difficult to interpret after treatment due to inflammation and necrosis in response to radiation, chemotherapy, or immunotherapy. False positive MRI due to treatment-related inflammation called “pseudo-progression” occurs frequently (7, 8) and makes clinical interpretation challenging (9). Given this limitation, there is a definitive need for improved, non-invasive methods for GBM diagnosis and monitoring. Ideally, such a method would just involve a simple blood draw.

Extracellular vesicles (EVs) are small plasma membrane-encapsulated particles released from all cells including GBMs and other cancer cells that can enter into the tumor microenvironment and bloodstream. Their cargo reflects their cell of origin. Exosomes are small EVs (50–100 nm) of endocytic origin while microvesicles are larger particles (100–1,000 nm) shed via direct cell membrane budding. EVs contain proteins (tumor antigens, immunosuppressive, and/or angiogenic molecules) and nucleic acids (microRNAs, mRNA) specific to cancer cells (10, 11), suggesting a role in intercellular communication between tumors and other cells (9, 11–13). Exosomes specifically contain distinct small non-coding RNA species compared to microvesicles whose cargo more closely represents cytosolic contents (14, 15). Furthermore, patient-derived cell lines and patient plasma EVs contain brain tumor markers such as HER2, EGFRviii, and mutant isocitrate dehydrogenase 1 (IDH1) (9, 16, 17) and may contribute to suppressing the immune system (18–20). EVs are ubiquitous in body fluids including plasma, cerebral spinal fluid (CSF), aqueous humor, amniotic fluid, saliva, synovial fluid, adipose tissue, and urine (21). Both plasma and CSF EVs including exosomes have been proposed as a source of biomarkers for liquid biopsies in GBM patients (22, 23). By analyzing EV cargo, it may be possible to track and predict tumor growth and allow early treatment for patients whose exosome composition correlates with tumor progression. Alternatively, patients with treatment-related pseudo-progression may be spared unnecessary and potentially ineffective changes in treatment strategy.

Some of the potential strengths of this approach lie in its non-invasiveness and simplicity. There have been some studies utilizing GBM patients' plasma EVs (24) but more have relied on CSF (22, 25, 26). Potential advantages of CSF over plasma include absence of contaminating plasma proteins and fewer contaminating non-tumor EVs. Furthermore, many studies of GBM patients' blood and CSF EVs have relied on complex technologies to separate tumor-derived EVs from the multitude of other EVs and proteins such as chip-based or droplet digital PCR analysis (25, 26). These studies also rely on purifying or amplifying for expression of known glioma-derived molecules such as EGFRviii or R132H-mutant IDH1. Collectively these approaches highlight significant drawbacks for a purportedly non-invasive and simple test. While obtaining CSF by lumbar puncture is clearly less invasive than a brain biopsy, it remains substantially more invasive than a simple blood test and is unlikely to be viewed with enthusiasm by patients at their monthly follow up appointments. Furthermore, reliance on complex technologies based on expression of single tumor-associated molecules to isolate EVs may be a barrier both to widespread adoption of the technique and to widespread generalizability. A simpler, more inclusive technique suitable for analyzing plasma EVs in GBM patients would be ideal.

Several relatively simple techniques to isolate EVs from body fluids are currently employed, including ultrafiltration, size exclusion chromatography (SEC), flow field-flow fractionation (F4), sequential filtration, differential ultracentrifugation, density gradient ultracentrifugation (DGU), among others (27). Many initial studies of GBM EVs in body fluids focused on exosomes (<100 nm) as a particularly rich source of biomarkers (11, 24). The current gold standard to specifically isolate exosomes from cell culture is differential ultracentrifugation (low-speed centrifugation to remove cells and debris, high-speed ultracentrifugation to pellet exosomes) (28). Density gradient-based ultracentrifugation using sucrose or iodixanol (OptiPrep™) gradients has been reported to obtain more pure exosome preparations from cell culture supernatants that can be used for downstream “omics” profiling (29). However, it is not known whether similar techniques would yield highly pure exosome populations in GBM patients' plasma nor is it clear that bulk plasma exosomes would yield tumor-specific signatures without further purification of tumor-derived exosomes in some manner.

Therefore, we sought to develop a density gradient-based ultracentrifugation technique to isolate exosomes from GBM patients' plasma and to analyze these bulk plasma exosomes to determine if clearly measurable differences could be identified differentiating GBM patients from normal donors.

## Materials and Methods

### Patient Blood Collection and Plasma Isolation

**Figure 1:**
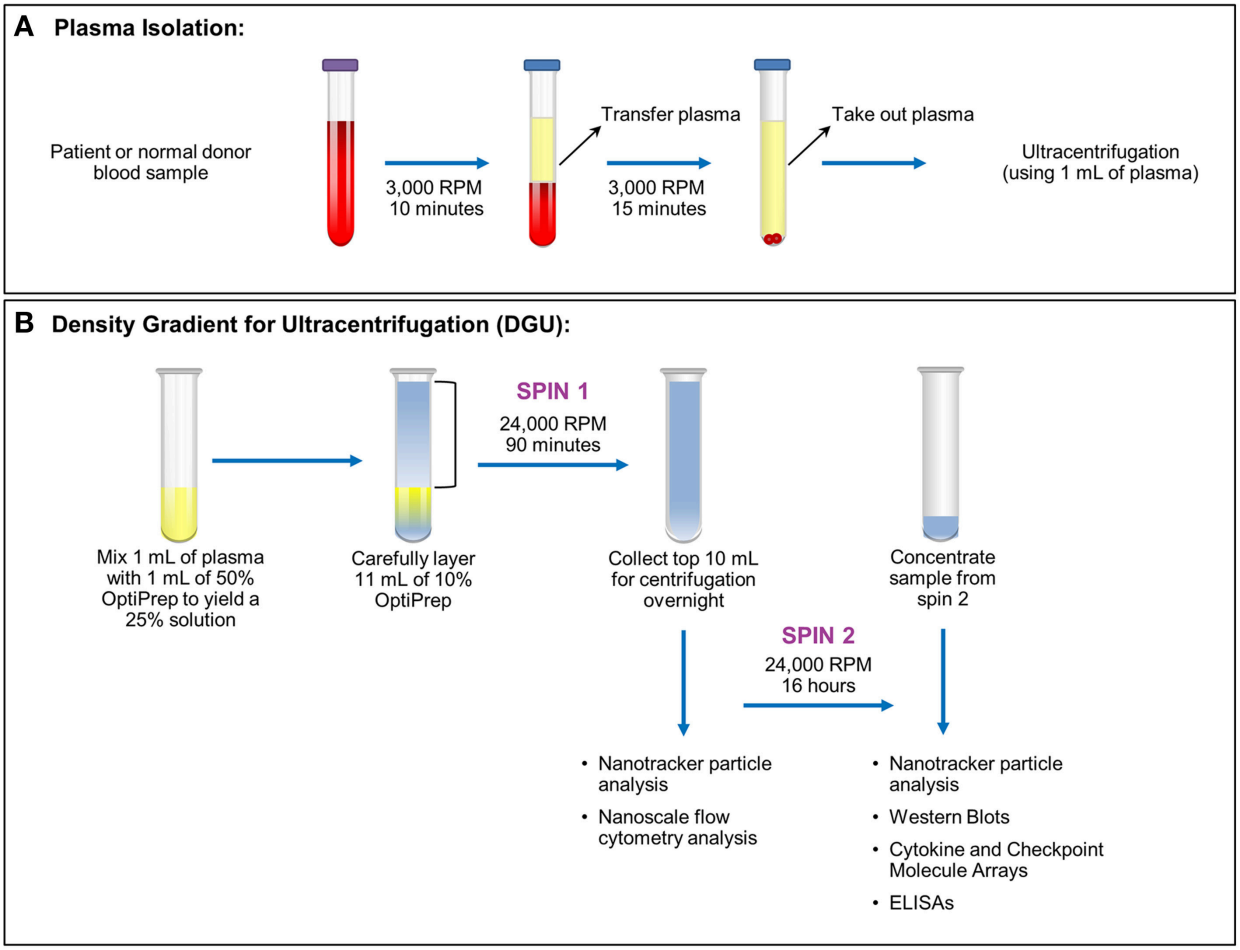
Plasma exosome isolation by density gradient ultracentrifugation (DGU). **(A)** Whole blood samples collected in EDTA tubes underwent brief centrifugation (3,000 RPM or 1,811 xg for 10 min) for plasma isolation. The isolated plasma was transferred to a fresh tube and spun for 15 min at 3,000 RPM (1,811 xg) to remove any remaining cellular debris and erythrocytes. **(B)**. Density gradient ultracentrifugation to purify exosomes was performed by mixing 1 ml of plasma with 1 ml of 50% OptiPrep solution. Eleven ml of 10% OptiPrep was layered on top of the homogenized solution and this underwent ultracentrifugation for 90 min at 24,000 RPM or 102,445 xg (Spin 1). The top layer (10 ml) was collected. A portion was used for nanotracker particle analysis and nanoscale flow cytometry analysis while the remainder underwent ultracentrifugation at 24,000 RPM (102,445 xg) for 16 h (Spin 2). The supernatant was discarded and the pellet was resuspended in a total volume of 200 μl. Further analysis (nanoparticle tracking, western blots, protein arrays, and ELISAs) was performed using this final, concentrated solution.

This was a Mayo Clinic Institutional Review Board (Mayo Clinic IRB# 15-006351) approved and Health Insurance Portability and Accountability Act (HIPAA) compliant study. Written informed consent was obtained from all patients. Nineteen samples were obtained from glioma patients undergoing surgery (6 females ages 35–67; 13 male ages 27–73). Nineteen anonymized control samples from normal donors were obtained through discarded material from the Mayo Clinic (Rochester) Blood Bank. All samples were acquired through collection of whole blood in ethylenediaminetetraacetic acid (EDTA) tubes. After blood collection, the samples were spun at 3,000 RPM or 1,811 xg (Eppendorf Centrifuge 5810 No. 0012529- rotor A-4-81) for 10 min. Plasma isolated from blood was then transferred into a 15 ml conical tube (Falcon No. 352097) and spun at 3,000 RPM (1,811 xg) for 15 min (Figure 1A). Plasma (1–9 ml) was recovered from each sample and stored in a sterile cryogenic vial (Corning Incorporated No. 430488) at −20°C.

### Isolation of Exosomes

Plasma exosomes were isolated by (DUG) (Figure 1B). Plasma (1 ml) was thawed and mixed in an ultra-clear centrifuge tube (Beckman Coulter No. 344060) with 1 ml of a 50% OptiPrep solution (45 ml of OptiPrep Density Gradient Medium, Sigma-Aldrich No. D1556, and 9 ml of OptiPrep diluent). The OptiPrep diluent preparation consists of 5 ml of 2.5 M sucrose, 0.1 g (6 mM) of EDTA, 1.08 g (120 mM) of Tricine at pH 7.8, and 45 ml of water. Eleven ml of 10% OptiPrep solution (4.4 ml of 50% OptiPrep solution, 17.6 ml of buffer A [100 ml of 2.5 M sucrose, 0.34 g (1 mM) of EDTA, 3.58 g (20 mM) of Tricine at pH 7.8, 900 ml of water]) were carefully layered onto the homogenized solution inside the tube. Samples were spun at 24,000 RPM (102,445 × g; Beckman Coulter Optima LE-80K Ultracentrifuge- rotor SW40 Ti No. 99U 10480) for 90 min. Nanotracker particle analysis using NanoSight (Malvern, NanoSight NS300) was performed using 10 μl from the bottom of the solution diluted into 1:100 in PBS. The top 10 ml of the solution were transferred to a new ultracentrifuge tube, and 1 ml was taken for nanoscale flow cytometry analysis. Samples were spun at 24,000 RPM (102,445 xg) for 16 h afterward. The supernatant was aspirated except for 200 μl. Samples from this EV-enriched 200 μl were utilized for western blots, cytokine and checkpoint molecules arrays, and ELISA assays. Nanotracker particle analysis was performed again to compare the particles obtained in both spins.

### Cytokine and Checkpoint Molecules Arrays

Pilot assays to evaluate Th1 and Th2 cytokines and checkpoint molecules that are fundamental to immune responses were performed in isolated plasma exosomes from 4 GBM patients and 4 normal donors using commercially available arrays per the manufacturer's instructions (Quantibody Human TH1/TH2 Array 1, RayBiotech No. QAH-TH-1; Quantibody Human Immune Checkpoint Molecule Array 1, RayBiotech No. QAH-ICM-1). These arrays measured the concentrations for the Th1 and Th2 cytokines IFN-γ, IL-10, IL-13, IL-2, IL-4, IL-5, IL-6, IL-8, GM-CSF, and TNF-α, and for the checkpoint molecules B7-1 (CD80), B7-2 (CD86), B7-H1 (PD-L1), B7-H2 (ICOS L), B7-H3 (CD276), CD28 (Tp44), CTLA-4 (CD152), ICOS (CD278), PD-1 (CD279), and PD-L2 (B7-DC). All plasma exosome samples were normalized to a protein concentration of 50 μg. Plasma exosomes (100 μl per sample) were loaded on the arrays and incubated at 4° overnight. Data extraction was performed by RayBiotech.

### Western Blot

Plasma exosomes were lysed in buffer (50 mmol/l NaCl, 50 mmol/l NaF, 50 mmol/l sodium pyrophosphate, 5 mmol/l EDTA, 5 mmol/l EGTA, 2 mmol/l Na3VO4, 1% Triton X-100, 0.5 mmol/l PMSF, 10 mmol/l HEPES, 10 μg/ml leupeptin at pH 7.4). Soluble protein extracts (20 μg per sample) were loaded into polyacrylamide gels (12.5%, BIO-RAD No. 3450015) and transferred onto PVDF membranes (BIO-RAD No. 162-0175). Membranes were incubated (1 h) in blocking buffer followed by overnight incubation with primary antibodies to PD-L1 (Cell Signaling No. 13684S), CD63 (Novus No. NB100-77913) and Flotillin-1 (Cell Signaling No. 3253S). After subsequent 1-h incubation with anti-rabbit (Jackson ImmunoResearch Laboratories No. 111-035-003) and anti-mouse (Jackson ImmunoResearch Laboratories No. 115-035-003) secondary antibodies, membranes were visualized by enhanced chemiluminescence.

### Nanoscale Flow Cytometry

The A50-Micro Nanoscale Flow Cytometer (Apogee Flow Systems Inc. No. S/N0105) was utilized to compare the total microparticles from GBM patients' and normal donors' unsorted whole plasma to exosomes samples isolated employing our DGU one-step protocol (exosome samples collected from spin 1–90 min). Whole plasma or exosomes isolated by DGU were diluted 1:40 in PBS to quantify microvesicle and exosome populations in each sample. Exosomes were defined as events <100 nm in size. Particles sizes were determined by using Apogee calibration bead mix (Catalog No. 1493) composed of 180, 240, 300, 590, 880, and 1,300 nm beads, and Apogee flow cytometer calibration beads (Catalog No. 1517) composed of 80 and 100 nm beads. The samples were measured in triplicates.

### Statistical Analysis

Statistical analysis was performed with GraphPad Prism software, using the two-tailed Student *T*-test. Statistical significance was determined at ^*^P < 0.05.

## Results

### DGU Isolates Exosomes From Normal Donors and Glioma Patients' Plasma

**Figure 2:**
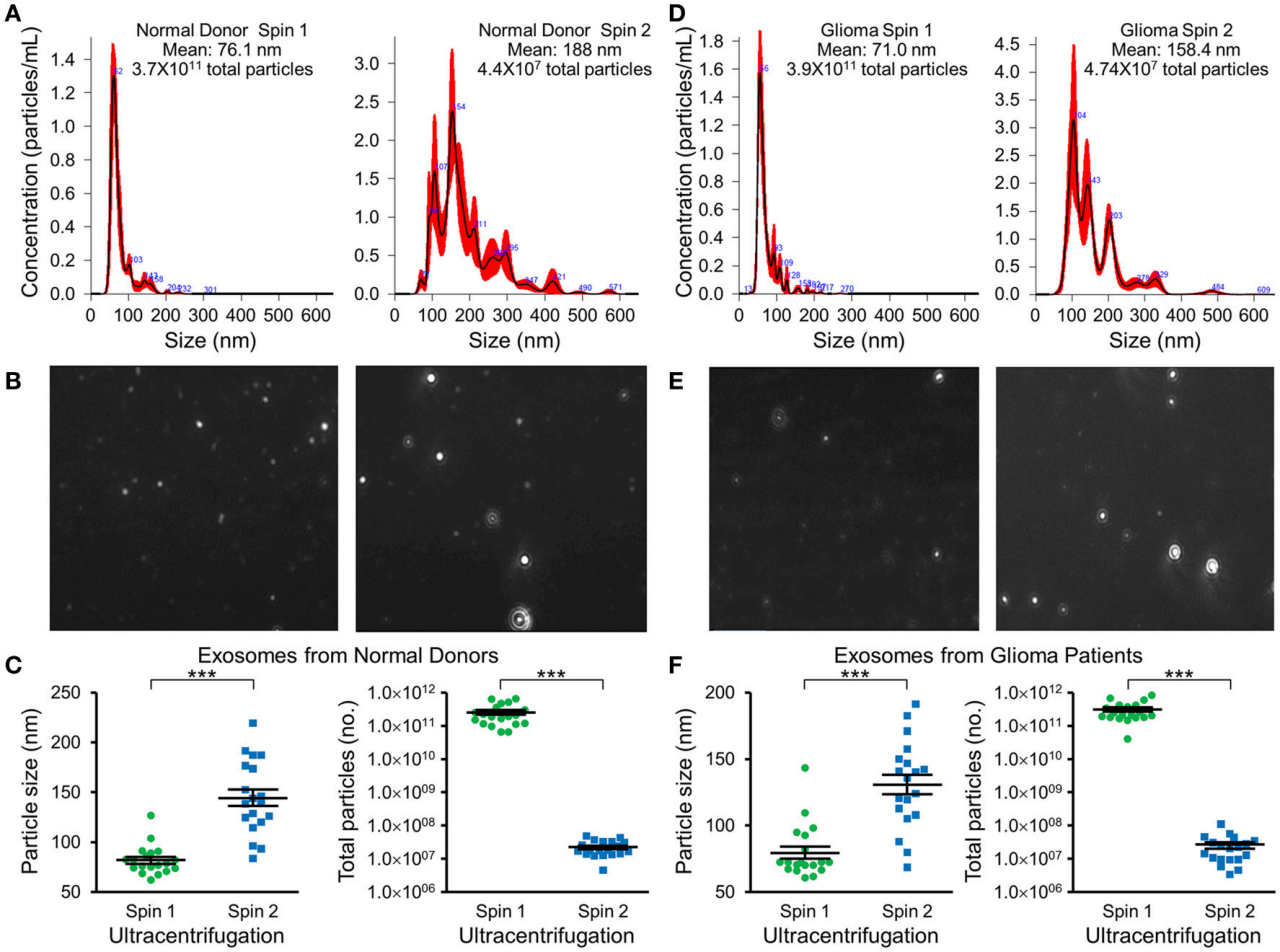
Impact of serial DGU on plasma exosome size and frequency. Representative nanoparticle tracker analysis histograms **(A,D)**, photomicrographs **(B,E)**, and pooled data for size and frequency (**C,F**; mean ± SEM, *n* = 20 per group, ***P < 0.001) of plasma extracellular vesicles after one density gradient ultracentrifugation (Spin 1) and two serial density gradient ultracentrifugations (Spin 2) for normal donors **(A–C)** and glioblastoma patients **(D–F)**. Note that for both normal donors and glioblastoma patients, extracellular vesicles isolated after a single ultracentrifugation (Spin 1) appear to be mostly <100 nm in diameter (i.e., exosomes). Performing two-step serial density gradient ultracentrifugation (Spin 2) results in particles that are both larger and less frequent. While this second spin is necessary to concentrate the samples for further molecular analysis, it appears to skew nanoparticle tracking results by causing aggregation of particles.

Nanotracker particle analysis determined the size and concentration of EVs isolated by DGU. One-step DGU (90 min) isolates a pure population of plasma exosomes quickly for nanotracker analysis. In contrast, the two-step DGU method takes an additional 16 h (90 min+ 16 h). It concentrates plasma exosomes efficiently for further analysis but results in exosome aggregation that skews nanotracker results (Figures 2A,D). Exosomes isolated by one-step DGU are physically smaller but more abundant than exosomes isolated by two-step DGU for both normal donors (Figures 2B,C) and glioma patients (Figures 2E,F; Supplementary Videos).

### IDH Wild-Type Glioma Patients' Plasma Exosomes Are Physically Smaller but Higher Concentration Than Normal Donors

**Figure 3:**
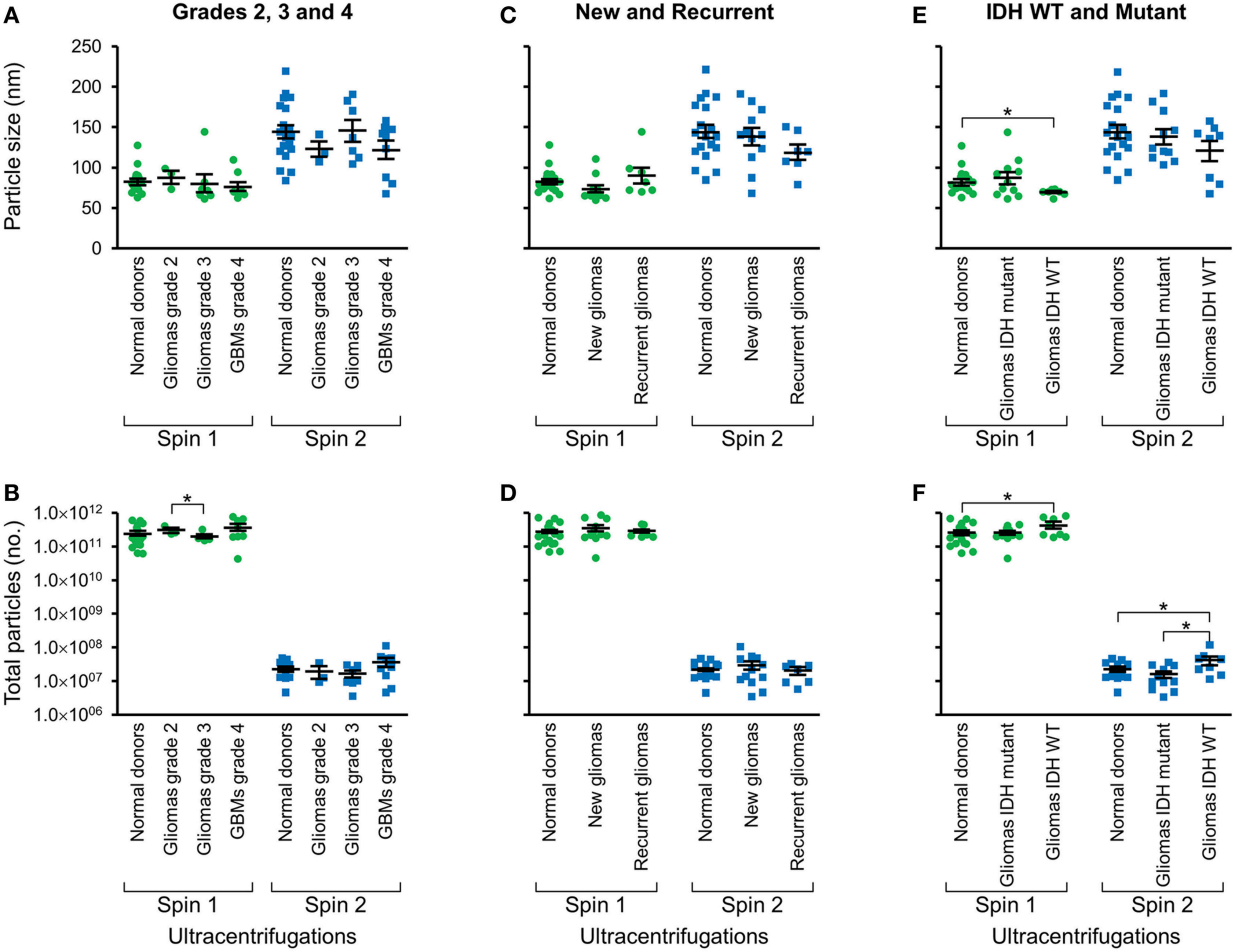
Glioma patient exosomes are grossly similar to normal donors. Pooled nanoparticle tracking analysis results comparing size **(A,C,E)** and frequency **(B,D,F)** following Spin 1 and Spin 2 in normal donor to different glioma grades **(A,B)**, newly diagnosed or recurrent gliomas **(C,D)** and IDH wild-type or IDH mutant gliomas **(E,F)**. *P < 0.05. Note that these results confirm increased size and decreased frequency of particles after Spin 2 compared to Spin 1 but show only mild differences between normal donors and glioma samples.

Following two-step DGU, nanotracker analysis to determine size and concentration was also performed on exosomes isolated from glioma tumors that were grade 2, 3, or GBM grade 4 (Figures 3A,B), new or recurrent (Figures 3C,D), and IDH WT or mutant (Figures 3E,F). No significant differences in size or concentration were observed between one or two-step DGU for grade 2, 3, or 4, or between new or recurrent tumor patient plasma exosomes (Figures 3A–D). However, one-step DGU demonstrated that IDH WT patients' plasma exosomes are significantly smaller but more abundant than normal donors (Figures 3E,F).

### Decreased IFN-γ, IL-10, and IL-13 Concentration in GBM Patients' Plasma Exosomes

**Figure 4:**
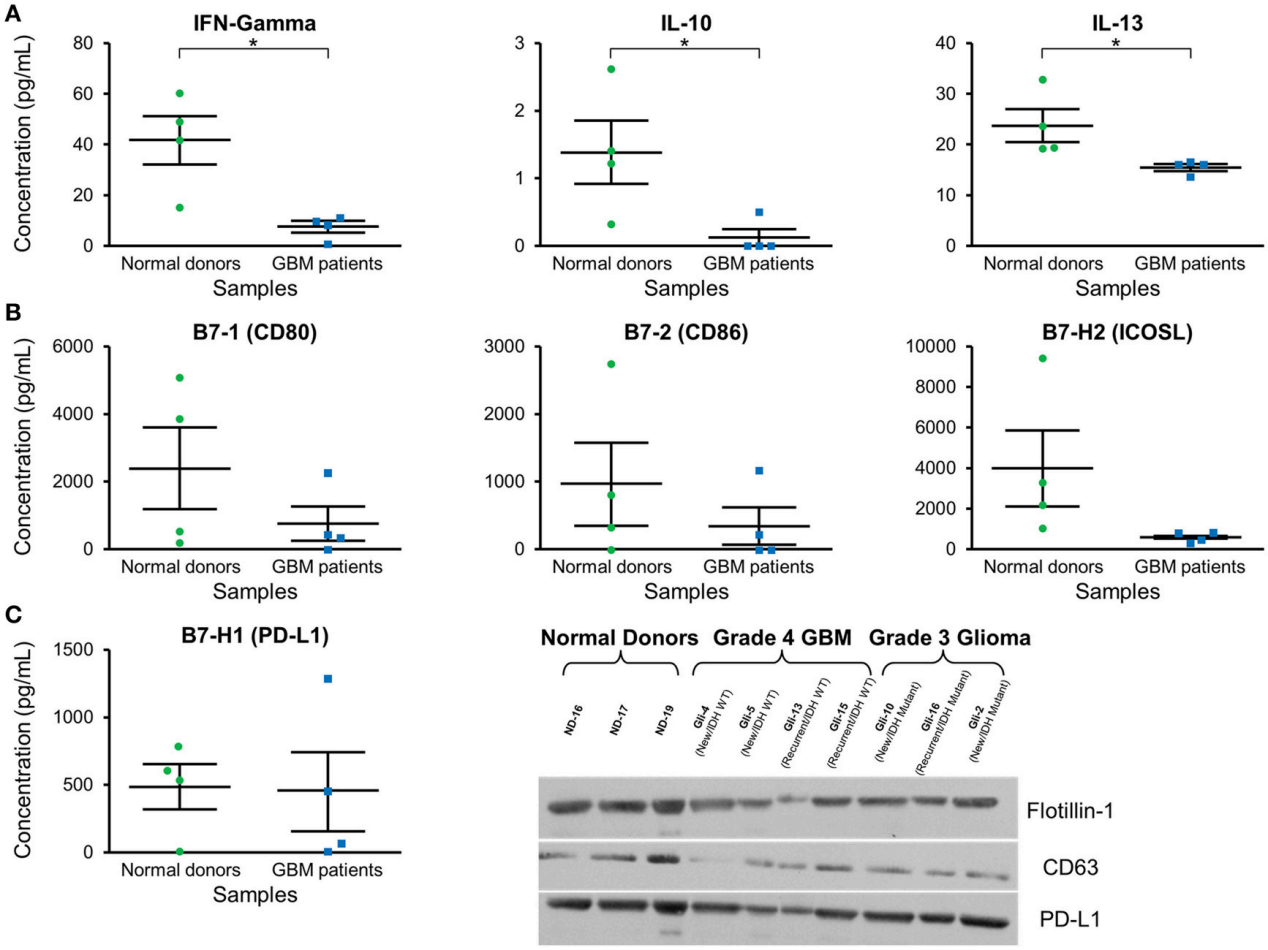
Cytokine and checkpoint molecule arrays from plasma exosomes showed a decreased concentration of IFN-γ, IL-10, IL-13, B7-1, B7-2, and ICOSL in GBM patients in comparison to normal donors. **(A)** Quantification of IFN-γ, IL-10, and IL-13 in plasma exosomes from normal donors and grade 4 GBM patients' plasma exosomes (mean ± SEM, *n* = 4/group, *P < 0.05). **(B)** Quantification of B7-1, B7-2, and ICOSL in plasma exosomes from normal donors and grade 4 GBM patients' plasma exosomes (mean ± SEM, *n* = 4/group). **(C)** Quantification of the immunosuppressive checkpoint and T cell costimulatory homolog protein PD-L1 in plasma exosomes from normal donors and grade 4 GBM patients' plasma exosomes (mean ± SEM, *n* = 4/group). Interestingly, PD-L1 is found at similar levels in both normal donor and glioma patient plasma exosomes and these findings were confirmed by western blot. Western Blot analysis also shows that exosomal markers Flotillin-1 and CD63 are found universally in plasma exosomes from normal donors (*n* = 3), glioblastoma (grade 4) patients (*n* = 4), and grade 3 glioma patients (*n* = 3), though CD63 expression may be slightly reduced in glioma patients.

Cytokine expression was evaluated in plasma exosomes isolated from grade 4 GBM patients and normal donors (Supplementary Table 1). We found a significantly decreased concentration of the cytokines IFN-γ, IL-10, and IL-3 in GBM patients' plasma exosomes (Figure 4A). IFN-γ ELISA showed similar but less pronounced results (*p* = NS; data not shown).

### Decreased Concentration of Co-stimulatory B7-1, B7-2, and ICOSL in GBM Patients' Exosomes, but Similar Levels of Programmed Death-Ligand 1 (PD-L) as Normal Donors

Checkpoint and costimulatory molecule expression was evaluated in plasma exosomes isolated from grade 4 GBM patients and normal donors (Supplementary Table 2). Trends toward decreased expression were observed for CD80, CD86, and ICOSL in GBM patients (*p* = NS; Figure 4B). Interestingly, immunosuppressive PD-L1 expression was the same between normal donors and GBM patients (Figure 4C) by both protein array and western blot.

### Exosome Markers CD63 and Flotillin-1 Are Present in Plasma Exosomes

Western blots were employed to demonstrate the expression of the exosome markers CD63 and Flotillin-1 in glioma patients' and normal donors' plasma exosomes isolated by two-step DGU. Both CD63 and Flotillin-1 were detected in normal donors, grade 4 GBM and grade 3 glioma patients' exosomes, indicating again that our DGU protocol allows the isolation of exosomes (Figure 4C).

### Nanoscale Flow Cytometry Analysis Detected a Pure Population of Plasma-Derived Exosomes Isolated by DGU

**Figure 5:**
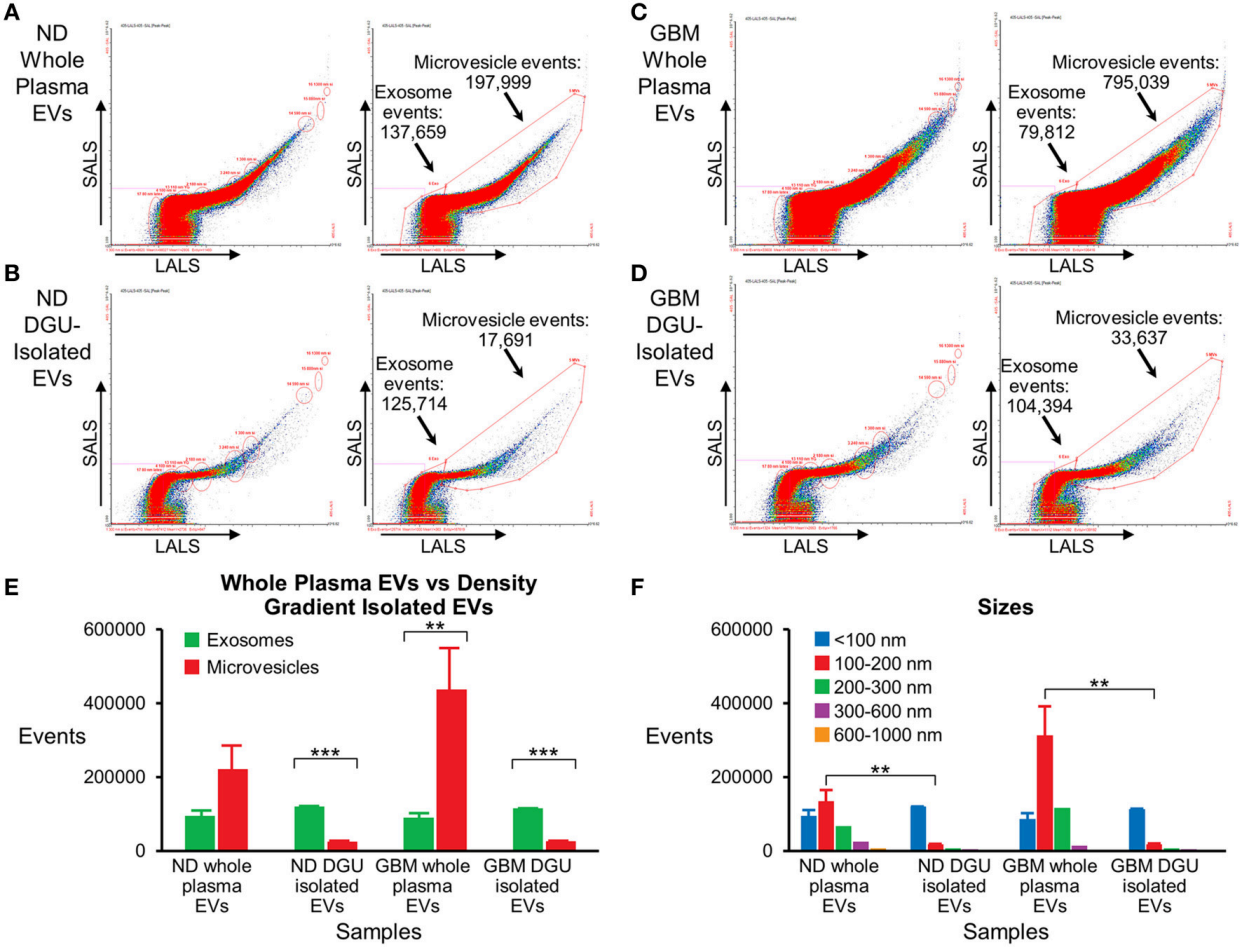
Nanoscale flow cytometry confirms plasma exosome purification by serial DGU. An enriched exosomes population identified in whole plasma particles and exosomes isolated by one-step DGU and analyzed with nanoscale flow cytometry (Short-angle light scatter-SALS vs. long-angle light scatter-LALS). Nanoscale flow cytometry representation of one normal donor **(A)** and one GBM **(C)** whole plasma EVs, illustrating exosome and microvesicle events. Nanoscale flow cytometry representation of one normal donor **(B)** and one GBM **(D)** isolated EVs using our one-step DGU protocol. An enriched population of exosomes and a decreased in the microvesicle population was observed. **(E)** Quantification of exosomes and microvesicles from whole plasma EVs-vs.-density gradient isolated EVs, confirming enrichment of exosomes and decreased in microvesicles using our DGU protocol. An increase in microvesicles was observed in GBM whole plasma EVs in comparison to ND whole plasma EVs. **(F)** Particle sizes of whole plasma and isolated EVs using our DGU method. An enriched population of particles that were <100 nm (exosomes) was observed employing our DGU method, whereas particles that were 100–1,000 nm (microvesicles) were reduced. In GBM whole plasma, particles that were 100–200 nm (microvesicles) were enriched in comparison to ND whole plasma (mean ± SEM, *n* = 3/group, ***P < 0.001, **P < 0.01).

Nanoscale flow cytometry analysis demonstrates that plasma exosomes are enriched in both normal donors (Figures 5A,B,E) and GBM patients (Figures 5C,D,E) after DGU. Interestingly, it also demonstrates increased microvesicles (100–1,000 nm) in GBM patients' whole plasma prior to DGU compared to normal donors (Figures 5A,C,E). Particle size was assessed using calibration beads further demonstrating that our method enriches for exosomes (<100 nm) instead of microvesicles (100–1,000 nm), but there are increased in microvesicles (100–200 nm) in GBM patients' whole plasma (Figure 5F).

## Discussion

Our findings demonstrate that DGU is a practical method to isolate plasma exosomes in GBM patients. A single 90-min DGU efficiently isolates exosomes for nanoparticle tracking which demonstrates that plasma exosomes from patients with IDH wild type tumors [generally more aggressive with decreased overall survival compared with IDH mutant tumors (30, 31)] are smaller but more numerous than normal donors. Most (90%) IDH mutations in gliomas represent a single mutation in IDH1 (R132H) but a small number of additional non-canonical IDH1/IDH2 mutations occur. It is possible that these additional mutations could be associated with different plasma exosome findings but their low frequency in our small sample precludes meaningful analysis. Nevertheless, differences in the size and frequency of plasma exosomes in patients with genetically more aggressive (IDH WT) gliomas compared to normal donors underscores their potential to reflect tumor burden and, possibly, tumor biology.

To explore this further, we found that an additional 16-h DGU concentrated exosomes efficiently for cargo analysis by western blot and protein array though it also resulted in exosome aggregation that skewed nanoparticle tracking. Furthermore, our pilot data suggested decreased cytokine and co-stimulatory marker expression in GBM patients' plasma exosomes compared to normal donors. Importantly, we did not see increased immunosuppressive molecules that are expressed by GBM cells in GBM patients' plasma exosomes. This may reflect global systemic immunosuppression seen in GBM patients (32–34), likely as a “whole organism response” rather than a specific signature of tumor-derived GBM exosomes that are presumably diluted in plasma by exosomes from many other sources. Further studies are required to determine if this reflects tumor burden. Nevertheless, plasma exosomes in GBM patients clearly have distinct cargo from healthy donors and could be a rich source of tumor-associated biomarkers.

Surprisingly, immunosuppressive PD-L1 was highly expressed in plasma exosomes from both GBM patients and normal donors. GBM-EV PD-L1 expression has been reported by others (35) and been demonstrated *in vitro* in our hands (data not shown). While this may well have immunosuppressive consequences in the tumor microenvironment or elsewhere, our data suggest that the mere presence of PD-L1 in plasma exosomes is not necessarily indicative of abnormal immunosuppression as it is ubiquitous in normal donors. Whether exosomal PD-L1 in concert with other tumor-derived molecules or PD-L1 expression by other non-exosomal EV compartments has more immunological significance remains to be seen.

We have demonstrated a simple, effective method for enriching plasma exosomes suitable for biomarker analysis but it is not yet clear whether our technique is the optimal method. For example, some standard methods for EV isolation like differential centrifugation can enrich for particular EV sizes by multiple centrifugation steps to pellet cells (300xg), microvesicles (10,000xg), and exosomes (100,000xg) (36). Others comparing multiple exosome isolation techniques found that DGU isolation provides the highest purification of exosomes from conditioned media, was comparable to SEC when evaluating protein exosome markers, but was slower than serial ultrafiltration (28). Others have found that DGU outperformed both ultracentrifugation and commercially available ExoQuick and Total Exosome Isolation precipitation for purifying cell culture exosomes (29). However, less has been published about exosomal isolation from body fluids by DGU. Furthermore, specimen handling, appropriate controls, and isolation and analysis techniques have not been standardized (21, 37).

Like most investigators initially (11, 24), we focused on purifying exosomes as these seemed the most likely to yield tumor-specific biomarkers. While we demonstrated preliminary evidence that GBM patients' plasma exosomes are distinct in size, frequency, and cargo from normal donors, we also showed that the largest difference between GBM patients' and normal donors' EVs is actually in microvesicle concentration. This suggests that microvesicles (which are specifically excluded by our exosomal purification) may also be critical sources for plasma biomarkers. Indeed, others have begun exploring this possibility (14, 38), though primarily in tumor-derived but not bulk plasma microvesicles.

In summary, we have demonstrated a simple method to isolate plasma exosomes, highlighted differences in size and frequency in plasma EVs between GBM patients and normal donors, and presented evidence for decreased expression of inflammatory markers in GBM patients' exosomes compatible with the effects of tumor-mediated immunosuppression. This suggests that plasma EVs may be a rich source of biomarkers that could form the basis of a “liquid biopsy” for GBM. We plan to pursue more comprehensive analyses by RNA-arrays, RNA sequencing, proteomics, and/or metabolomics to identify and validate candidate genes in GBM plasma EVs that correspond with tumor burden and response to therapy. Finally, the exosome isolation method employed here yields a pure population of exosomes. This will facilitate obtaining reliable “omics” data and identifying exosome-specific functions and biomarkers (29) from plasma.

## Data Availability

All datasets generated for this study are included in the manuscript and/or the Supplementary Files.

## Ethics Statement

This study was carried out in accordance with the recommendations of the Mayo Clinic Institutional Review Board (IRB# 15-006351), with written informed consent from all subjects. All subjects gave written informed consent in accordance with the Declaration of Helsinki. Control samples from normal donors were obtained through anonymized, discarded material from the Mayo Clinic (Rochester) Blood Bank. The protocol was approved by the Mayo Clinic Institutional Review Board.

## Author Contributions

IP provided direction and made revisions to the manuscript. LC performed all experiments, provided the figures, and wrote the manuscript. TP assisted with some of the experiments and optimization of the isolation technique. MC assisted with the Nanoscale Flow Cytometry experiments and provided figures from these experiments. AJ provided direction. All authors approved the manuscript.

### Conflict of Interest Statement

The authors declare that the research was conducted in the absence of any commercial or financial relationships that could be construed as a potential conflict of interest.
