# Supplementary material for: Isolation and Analysis of Plasma-Derived Exosomes in Patients With Glioma
Source: Front Oncol. 2019 Jul 16;9:651. doi: 10.3389/fonc.2019.00651 (PMC6646733; doi:10.3389/fonc.2019.00651)
Supplement: Supplementary file 3 [file Data_Sheet_1.PDF]

Supplementary Tables with Legends

Table 1. Cytokines source, concentration, and p-value analyzed by cytokine arrays. Concentrations of the Th1 and Th2 cytokines IFN-γ, IL-10, IL-13, IL-2, IL-4, IL-5, IL-6, IL-8, GM-CSF, and TNF-α from normal donors and GBM plasma exosomes. A decreased in the expression of IFN-γ, IL-10, and IL-13 cytokines were observed in GBM patients in comparison to normal donors.

Cytokines	Source	Concentration (pg/mL)	P
IFN-γ	ND	41.38±19.13	**0.013**
	GBM	6.880±4.770	
IL-10	ND	1.396±0.9394	**0.040**
	GBM	0.1259±0.2519	
IL-13	ND	23.84±6.368	**0.045**
	GBM	15.61±1.291	
IL-2	ND	25.62±5.247	0.266
	GBM	22.32±1.176	
IL-4	ND	–	–
	GBM	–	
IL-5	ND	8.339±1.317	0.360
	GBM	7.650±0.4464	
IL-6	ND	–	–
	GBM	–	
IL-8	ND	17.09±8.236	0.059
	GBM	6.958±2.810	
GM-CSF	ND	9.091±6.420	0.300
	GBM	4.882±3.729	
TNF-α	ND	16.53±25.58	–
	GBM	–	

Table 2. Costimulatory and checkpoint molecule source, concentration, and p-value analyzed by cytokine arrays. Concentrations for the checkpoint molecules B7-1 (CD80), B7-2 (CD86), B7-H1 (PD-L1), B7-H2 (ICOS L), B7-H3 (CD276), CD28 (Tp44), CTLA-4 (CD152), ICOS (CD278), PD-1 (CD279), and PD-L2 (B7-DC) from normal donors and GBM plasma exosomes. A decreased in the expression of B7-1 (CD80), B7-2 (CD86) and B7-H2 (ICOS L) was observed in GBM patients in comparison to normal donors, but these were not statistically significant. PD-L1 expression was similar between normal donors and GBM patients, confirming our western blot results for PD-L1 expression.

Checkpoint molecules	Source	Concentration (pg/mL)	P
B7-1 (CD80)	ND<br>GBM	2408±2418<br>763.3±1025	0.257
B7-2 (CD86)	ND<br>GBM	973.4±1225<br>348.3±555.6	0.390
B7-H1 (PD-L1)	ND<br>GBM	475.4±334.2<br>444.7±587.0	0.931
B7-H2 (ICOSL)	ND<br>GBM	4016±3739<br>628.8±256.2	0.121
B7-H3 (CD276)	ND<br>GBM	–<br>–	–
CD28 (Tp44)	ND<br>GBM	175.6±129.8<br>65.03±130.1	0.274
CTLA-4 (CD152)	ND<br>GBM	–<br>–	–
ICOS (CD278)	ND<br>GBM	1510±184.1<br>1406±464.5	0.691
PD-1 (CD279)	ND<br>GBM	62.83±73.36<br>95.73±191.5	0.760
PD-L2 (B7-DC)	ND<br>GBM	990.6±187.5<br>1132±435.1	0.572

Supplementary Video Legends

Video 1. Visualization of plasma-derived exosomes isolated by one-step DGU. Nanotracker particle analysis was performed using 10 µl of the plasma GBM sample from spin 1 in 990 µl of PBS (1:100) to determine size and concentration of particles. This analysis suggests that exosomes isolated by one-step DGU are physically smaller but more abundant than exosomes isolated by two-step DGU.

Video 2. Visualization of plasma-derived exosomes isolated by two-step DGU. Nanotracker particle analysis was performed using 10 µl of the plasma GBM sample from spin 2 in 990 µl of PBS to determine size and concentration of particles. This analysis suggests that exosomes isolated by two-step DGU are physically larger but less abundant than exosomes isolated by one-step DGU.
